# Khuluma: Using Participatory, Peer-Led and Digital Methods to Deliver Psychosocial Support to Young People Living With HIV in South Africa

**DOI:** 10.3389/frph.2021.687677

**Published:** 2021-07-15

**Authors:** Millicent Atujuna, Nikita Simpson, Malebo Ngobeni, Tebogo Monese, Danielle Giovenco, Carey Pike, Zuzana Figerova, Maretha Visser, Maurice Biriotti, Anna Kydd, Linda-Gail Bekker

**Affiliations:** ^1^Desmond Tutu HIV Centre, Cape Town, South Africa; ^2^SHM Foundation, London, United Kingdom; ^3^Department of Anthropology, London School of Economics and Political Science, London, United Kingdom; ^4^Department of Epidemiology, The University of North Carolina at Chapel Hill, Chapel Hill, NC, United States; ^5^Department of Psychology, University of Pretoria, Pretoria, South Africa

**Keywords:** mHealth interventions, mobile phone, peer-to-peer support, adolescents, South Africa

## Abstract

Khuluma is a psychosocial and peer-to-peer mHealth intervention that uses text messaging to facilitate support groups for adolescents living with HIV (ALWH) with the aim of contributing toward positive health outcomes. Although use of mobile technology in the form of mHealth interventions has proliferated recently in the field of health, published literature describing methods and processes of its application are limited. We present a set of methods and processes utilised to develop and pilot the Khuluma mHealth intervention amongst young people (15–20 years) in South Africa. We recruited and enrolled 52 adolescents (15–20-year olds) from four clinics in Pretoria and Cape Town to participate in a 6-month pilot of Khuluma. Participants were ALWH, aware of their status, on antiretroviral therapy for more than 12 months, and not suffering from severe depression. We conducted four pre and post intervention focus group discussions (FGDs) with a proportion of ALWH (*n* = 36) enrolled in the pilot study using participatory methods. Several processes were utilised to then implement this pilot study. These included engaging ALWH for minor study implementation modifications; forming virtual groups; activating the mHealth platform; facilitating and delivering the Khuluma intervention. The acceptability of the intervention was informed by follow-up focus group discussions and text message data. The initial participatory processes helped to tailor the intervention design to participants' needs. The peer-led facilitation of the groups allowed for the provision of sensitive psychosocial support that allowed young people to express themselves freely, develop a sense of self-worth, and interact more. The nature of the mobile technology also allowed participants to build friendships beyond their geographic area and interact with their peers in real time. Within the evolving context of COVID-19, establishing evidence-based processes and methods for intervention design and curation in virtual spaces is critical.

## Introduction

Advances in the functionality, speed, and availability of mobile devices and networks, coupled with the increased penetration of mobile phones especially among young people (1–3), has garnered interest in the utilisation of mobile phones in health service delivery (4–8). Known as mHealth, this field involves the use of mobile phones and other mobile devices to improve health knowledge, behaviours, and outcomes (9, 10). Within the evolving context of the COVID-19 pandemic, establishing evidence-based processes and methods for design and implementation of mHealth technology is critically needed.

Globally, trials and feasibility studies have shown the effectiveness of mHealth interventions in targeting a variety of health conditions including HIV prevention and treatment, non-communicable diseases, mental health and TB in high and low-income settings. These interventions provide health information, e-reminders, generate awareness, monitor medication adherence, and facilitate consultation and even diagnosis (11, 12), while utilising voice messaging, text messaging (SMS), multi-media messaging, software for easy data collection and gamification (6–8). There has concurrently been an increased focus on the use of mHealth for adolescent Sexual Reproductive Health (SRH) challenges, to optimise the ways in which mHealth can improve access to SRH information and knowledge and overcome barriers such as provider bias and stigma experienced by adolescents in traditional health facilities. In addition, mHealth provides a confidential safe space within which adolescents can freely express themselves (13–15).

We are also seeing increased use of mHealth in primary health care, HIV, malaria, mental health, and child care (4, 16, 17) on the continent. To that end, national governments and ministries of health are increasingly recognising the potential of mHealth in delivering quality health services affordably, enabling differentiated care, and information to less accessible districts (16). There is a call for the development of strategic frameworks on the use mHealth in the face of limited evidence on scale up and implementation (18).

In South Africa (SA), mHealth has been seen as an important accelerator of universal health coverage (19), as mobile phone ownership and connectivity is increasing even in low resource and rural settings (15). With the largest HIV anti-retroviral Therapy (ART) programme, mHealth innovation in SA has been leveraged for patient retention and medication adherence challenges, and in improving uptake of early HIV testing amongst men (20). mHealth tools have also been used to improve antenatal and postnatal care for pregnant women and new mothers (11, 21, 22). mHealth has enhanced uptake of family planning services (4) chronic medication provision and supporting patients (22) with mental health conditions (23). More recently, the COVID-19 pandemic has forced healthcare workers to use mHealth to conduct tele-consultations, Covid-19 screening and delivery of results, COVID-19 vaccination, and for broad dissemination of health information (24, 25).

To date, however, mHealth interventions have commonly used one-to-one communication, engaging the participant individually *via* their mobile phone. Such interventions lack the level of facilitation, personalization, and intimacy that people may need to address personal challenges and psychosocial barriers to uptake and adherence (10, 26). Although mainly conducted in high income settings and with adult populations, there are examples of expanded use of mHealth tools to group settings, with potential benefits for isolated or mentally distressed populations (27–29). There is thus a significant opportunity to leverage group mobile phone technology to expand the reach and exploit peer support groups and peer-led education.

In-person peer support groups are an important and powerful mechanism for addressing clinical outcomes and well-being in the field of HIV (30–32). For adolescents living with HIV (ALWH), the reciprocal nature of peer groups, supporters share their knowledge through lived experiences, provide hope, emotional, social, and instrumental support to their peers (33–36), while simultaneously gaining comfort, strength, and healing from their own disclosures (36). The similarity in experiences between the peer leaders and beneficiaries allows discussion of common challenges and provides opportunities for support (5, 37–40).

Several randomised control trails (RCTs), observational, and qualitative studies conducted in sub-Saharan Africa provide evidence suggesting that in-person peer support groups are effective in improving HIV testing, linkage to care, adherence, retention, and viral suppression, primarily by increasing confidence, self-esteem, and reducing the effects of stigma (20, 25, 26, 28–34, 41–44). Yet, there are a number of barriers to in-person program participation, including logistical challenges such as transportation and child care, human and infrastructure resources, and privacy (42, 43). These challenges are exacerbated by COVID-19 social distancing regulations. Reports of increased loneliness and anxiety following the closure of schools, clinics, and clubs and limited visitation of friends and family during extended lockdown periods have increased (45). The closure of adolescent support or adherence clubs has prevented regular in-person interaction between adolescents and healthcare professionals. Mobile technology based-peer led support, designed using participatory and human-centred approaches, can provide a cost-effective alternative to mitigate these barriers (39).

In this paper we describe the design and delivery of Khuluma, a group-based mHealth intervention, to provide psychosocial support groups to ALWH through the use of peer-led facilitation.

## Methods and Materials

### The Intervention Model: Project Khuluma

The Khuluma intervention model was trialled among new mothers in Mexico in 2008, and has been refined, adapted and replicated in Guatemala (46), the UK, Zimbabwe, Zambia, and SA (47), with prior evaluation of the model showing positive health and mental health outcomes for populations experiencing stigma and social isolation (47).

The model was first adapted to meet the needs of ALWH in Pretoria, SA in 2013 with additional iterations made to the model using data obtained from interviews, FGDs and small Khuluma support groups that were implemented between 2013 and 2015. Overall, the intervention adaptation involved three steps. Firstly, a needs assessment, which included a series of semi-structured individual interviews with Health Care Professionals (HCPs) and ALWH (13–18 year olds) and further FGDs with ALWH, was conducted to determine challenges associated with treatment uptake, key issues facing young people beyond the clinic, modes of communication and gender norms, and their use of technology. Secondly, these insights were revisited by a group of key stakeholders (including HCPs, researchers and ALWH) through a participatory process of adaptation. The third and final phase engaged young people (some of whom had taken part in Khuluma groups) in a participatory action research workshop that used artistic methods to create a democratic and “safe space” (48, 49), wherein stakeholders disclosed their personal needs, experiences, and ideas of what they wanted from a support system to facilitators. The discussions identified what forms of support the youth lacked, what kind of support they hoped to get from an intervention, and their acceptance and understanding of using of mobile phones to receive support *via* text messages. The discussions highlighted adolescents' needs around social support, stigma, barriers, and facilitators to accessing health care, medical adherence, and mobile technology access and usage. It also elicited insights into recruitment, group facilitation, ideal group sizes, training needs, parental consent and assent, and the adolescent context around school attendance, clinic attendance, and ideal times for session group discussions.

The adapted “Khuluma” intervention, which means “talk/speak” in Zulu, was piloted among ALWH in Cape Town and Pretoria in 2016, using SMS technology and feature phones. At the time of this pilot study, the intervention model was a time-bounded social support program designed to run for 3 months, wherein participants were placed in support groups of 8–15 peers who shared experiences of living with HIV. These support groups were virtually enabled through a digital platform, where participants discuss—peer-to-peer, at anytime, anywhere *via* text message—a range of issues pertinent to their needs. Active facilitation of group sessions utilising the Khuluma curriculum was held Monday to Friday from 3 to 7 p.m. Critically, the model was peer-led, with trained peer mentors facilitating conversations, supported at all times by trained professional counsellors. Participants chose a pseudonym, allowing them to remain anonymous and communicate in small groups. Conversations were externally monitored three times per day by trained staff.

### Study Design and Setting

The data used in this paper was drawn from Project Khuluma, an mHealth pilot intervention study that deployed virtually facilitated peer to peer psychosocial support groups *via* mobile phone to enhance connectivity and improve well-being among ALWH aged 15–20 years in South Africa. This 6-month study employed a pretest-posttest design to assess acceptability and feasibility of the model, as delivered using mobile phone SMS messaging. Peer-to-peer psychosocial support groups ran for 3 months, while the total pilot study ran for 6 months including the time for recruitment and evaluation. A behavioural assessment survey (whose outcomes are not reported here) and focus group discussion were conducted at baseline and at 6 months after enrolment into the intervention.

The study was conducted in four adolescent HIV clinics located in both Pretoria—Steve Biko Hospital and Kalafong Hospital—and Cape Town—KidzPositive clinic located in Groot Schuur Hospital and the Hannan CrusAID adolescent clinic in Gugulethu. Three of the selected clinics were located in an urban or peri-urban setting, although patients attending these clinics came from urban, semi-urban, and informal communities. One clinic was located in an urban informal township. The majority of adolescents included in the study had perinatally acquired HIV, came from low socio-economic backgrounds, and had been receiving HIV care for a number of years.

### Study Sample

The study population consisted of 52 ALWH across the four clinics. Participants were aware of their status, 15–20 years-old, and on antiretroviral therapy for more than 12 months. Participants were screened for severe depression (according to a standard screening questionnaire) or other significant mental illness; and those with significant mental illness were excluded from the study and referred for care. A sub-sample of the enrolled population were purposively selected and invited to participate in four group discussions (FGDs). Two FGDs, comprising of seven participants each, were conducted at baseline, while two FGDs with 11 ALWH each were conducted at 6 months after study enrolment (36 participants in total).

### Steps and Processes of the Khuluma Pilot Study and Intervention Delivery

#### Participatory Adaptation Process and Strategies for Study Implementation

Prior to the implementation of the Khuluma pilot study, additional assessments were conducted using participatory engagement among 14 ALWH in Cape Town and Pretoria. These engagements were designed to understand: (1) ideal group sizes and composition, (2) technological requirements, (3) curriculum and facilitation strategy. The implementation of the pilot study involved four peer-to-peer support groups of 8–15 participants for 12 weeks, facilitated by a combination of expert professionals and peer mentors. It concluded with post-intervention group discussions and an analysis of the text message data generated.

#### mHealth Platform Using Text SMS Messaging System

All participants received a new feature phone that had SMS functionality (i.e., not a smart phone) and a registered SIM card to use during the intervention, to allow for data use monitoring and device security. Participants were given instructions on how to use the phone. Flickswitch^©^, was the software used to control SIM cards, to distribute and top-up text messages airtime (provided by Vodacom Foundation), and to track usage remotely. Only after enrolment were participants informed they would be allowed to keep the phone at the end of the project.

Group SMS messaging was facilitated by a short-code that allowed participants to send a message to a central number, from where it would be distributed to their group. The group SMS format was made possible with ZygoHubs^©^ and BulkSMS^©^ technology. ZygoHubs^©^ was a United Kingdom-based group text messaging platform that allowed several individuals to communicate *via* SMS using a single mobile phone number. For the purposes of this project, ZygoHubs^©^ assigned a mobile phone number to the support group and all of the messages sent to this designated phone number were delivered to each participant involved in the study *via* a web interface. BulkSMS^©^, a South African company, provided the application-to-person (A2P) messaging service necessary for mobile phones to access ZygoHubs^©^ software. The ZygoHubs^©^ web interface recorded all messages sent to the group mobile phone number from which investigators produced a transcript for analysis. Thus, we were able to provide a confidential SMS messaging system that allowed adolescents to text one another while maintaining anonymity. The system was designed to ensure that third party access was restricted with access codes only provided to participants, preventing family or friends from accessing messages without the participant's consent. All data transmitted from one phone to another were centrally archived.

#### Forming Virtual Groups

Participants were asked to decide on personal pseudonyms (“nicknames”) (example: Miracle, 2007). Beyond the pseudonyms, each participant was allocated a participant ID number (PTID) with which we used to identify the phone discussion. Four groups were allocated by the SHM Foundation team. The groups included both males and females, mixed participants across study sites, and were divided by age (two groups of 15–17 years and two of 18–20 years). Over the course of the 3-month period, some participants were moved between groups by facilitators dependent on their communication levels and preference of discussion topics. For example, if a participant was originally put in the group of 15–17 year olds, but wished to discuss topics of a sexual nature that their peers did not want to discuss, they were moved to the older age group. This meant that the number of participants in each group fluctuated but was never under 8 and never more than 15. In each group, we ensured a relatively equal mix of participants from Pretoria and Cape Town. Each group was assigned a trained peer mentor to facilitate the conversations.

#### Facilitation and Curriculum Delivery

The professional facilitators consisted of counsellors trained to work with ALWH, who had implemented the model previously and who had been trained in recruitment, facilitation and data collection processes. The professional facilitators were tasked with intermittent engagement in all groups, monitoring for problematic behaviour or conflict and identifying participants for referral. The peer mentors were ALWH from both provinces trained *via* a counsellor-led workshop on group facilitation and use of the technology platform. They were each assigned to one group.

The training workshop, which all facilitators attended prior to study implementation, was designed by the SHM Foundation, adapted from the Positive Connections manual developed by FHI360 for leading information and support groups for adolescents living with HIV (50). Facilitators were briefed on key issues facing this population, including HIV and AIDS knowledge, adherence to ART, stigma, disclosure, socioeconomic issues, gender-based violence and education. They were trained on facilitation skills and techniques such as active listening, empathetic encouragement, non-judgemental communication, asking open ended questions, validation and problem solving. They were encouraged to draw on their own experiences as parents, friends, siblings or caregivers in order to develop their skills. The facilitators were briefed on the model and technological platform. This involved imparting skills in trouble shooting and referral processes. The final part of the workshop involved scenario-based thinking, where participants were given difficult issues to engage and resolve that might occur in the groups, such as conflict between participants, non-participation, suicidal ideation or a participant spreading a myth.

Facilitators were given a training manual that included the subjects covered in this workshop. This training manual included a curriculum of text messages and suggested responses based on the co-developed list of topics as presented in Table 1 below.

**Table 1:** Curriculum script of potential text messages and excerpts of actual text message conversations.

**Curriculum topic: Understanding the difference between HIV and AIDS**			
**Curriculum script**		**Sample of actual text conversation between participants and facilitators**	
**Potential text message questions from ALWH**	**Potential facilitator response**	**Participant**	**Facilitator**
**“Will I die soon?”**	ALWH sometimes don't understand the difference between HIV and AIDS. They might think that a positive test result means they will die soon. You should tell them that with earlier detection, effective drug regimens and a healthy lifestyle, it is possible to remain alive and healthy. They also need to know that if they do not get treatment or if they do not properly adhere to treatment, then this could negatively impact on their health and make it more likely that their HIV will develop into AIDS. (**Khuluma Training Manual)**	**Dimpho**… “When i have hiv I am I going to die?”	“no you won't die Remember that there is treatment available to help you live a long happy productive life”
		**Dimpo… “I think I am going to die”**	“yes there is no cure for HIV but there are so many people that have been living with Hiv for many years. As long as you take your Medication as directed by the Doctor and also make sure that you discuss all your concerns with a doc”
		**Dimpho**… “If you don't take your medicine you are going to die”	(At this point the facilitator asks others to chime in. and the conversation continues)
**Curriculum topic 2: Stigma and disclosure**			
**Curriculum script**		**Sample of actual text conversation between participants and facilitators or from one participant to another in groups**	
**Potential text message questions from ALWH**	**Potential facilitator response**	**Participant**	**Response from other participants**
**“I am afraid that people will reject me, or be violent toward me. I can't tell anyone that I am HIV positive”**.	Adolescents living with HIV often experience stigma, discrimination and isolation. Acts of discrimination can range from inappropriate and hurtful comments to physical violence. Participants must be made aware of their rights. If however you become aware of serious incidents of discrimination or violence toward a participant, this must be reported to the team and may require intervention by appropriate authorities and professionals. **Or** With the high rates of stigma around HIV, ALHIV may be understandably weary of disclosing their status beyond their immediate family and healthcare professionals. Disclosure is not something you can decide on for an adolescent. Help by providing adolescents with a space in which they feel safe to share their experiences with one another. Assist them to weigh the risks and benefits of disclosure and point them in the direction of helpful resources and organisations who can offer further support on disclosure. **(Khuluma Training Manual)**	**Anonymous**… “I am afraid to tell my friends I am hiv, I can't tell my friends about my life”	“What I think is that I must get to know the person well before I can't tell her about my status and stuff” **(peer-to-peer text message conversation)**

Topics in the curriculum were categorised into four areas: (i) HIV knowledge, staying healthy and treatment adherence; (ii) Growing up, sex and relationships, and sexual health and positive prevention; (iii) Communication and problem solving, personal feelings, and decision making; (iv) Gender norms, stigma and discrimination, networks and social support. Each week, facilitators were given a number of topics to bring up in their groups. Every morning except on weekends, facilitators would check in with the group. Each weekday, after school hours, facilitators would bring up one of these topics. In the first weeks of the intervention, these topics were broadly about getting to know each other, but as the intervention progressed, sensitive topics were discussed as highlighted above. Facilitators were encouraged, however, to follow the lead of participants if they brought up a topic of interest.

### Data Collection

Prior to study enrolment, written informed consent for participants 18 years and older was obtained. Parental or guardian consent and assent was obtained from all participants under the age of 18 years. The consent forms were translated into the local language of the participant. This was followed by a behavioural assessment survey before engaging in intervention topics. During the intervention, data generated in the form of text group discussion messages were archived by our technology platform. Eighteen thousand two hundred and fifty-three text messages were generated and saved in an Excel document. Messages were already anonymised as participants used pseudonyms, but any further identifying features were removed to protect confidentiality. FGDs designed to understand intervention utility, acceptability, and feasibility were conducted in the local language by facilitators fluent in both English and local language spoken in each province. Notes were taken during the focus group discussion and later typed in a word document. Given that there were only 4 FGD transcripts, they were analysed without using coding software. The main themes in the exit focus groups discussions guide were: whether the participants found the platform easy to use, whether they enjoyed the discussions, what they liked or disliked about the platform, what they learnt, whether they would use this platform in future and what they would change to make it better.

The protocol, informed consent documents and tools were approved by the ethical review committees at the University of Cape Town (HREC 630/2014) and the University of Pretoria (GW20160616HS).

### Analysis

#### Analysis of Text Message Data

For the analysis of text message data, we utilised a grounded inductive analysis approach (51), involving a full review of each text message. Transcripts were distributed across a team of five researchers. Researchers initially read through transcripts of text messages to familiarise themselves with the data. Guided by the approach developed by Cutrona and Suhr (52) in their Social Support Typology, researchers coded each conversation (a series of messages sent on the same topic, on the same day) where they developed the initial codebook. They then met to agree on the codebook and coding framework. It was decided not to code each message due to the size of the database, the short form of the messages and the importance of capturing the dynamics of conversation, but rather focus on messages within conversation blocks. Researchers reviewed the transcripts of text messages again and coded the conversations according to the agreed coding framework. Through this approach, themes were refined and inconsistencies within themes corrected. Disagreements were resolved through group discussion. Exemplary quotes were isolated and charted under each theme. Text message conversations sent were already anonymised and as such attributions or demographic characteristics were not included. These attributions are therefore, not presented with participant quotes.

Finally, a workshop was held consisting of a multi-disciplinary team of technology specialists, sociologists, community members, literary scholars and linguists, epidemiologists, clinicians, the study implementation team at each site and three participants. Workshop attendees reviewed the themes and ensured that the example quotes were meaningful and accurately represented, based upon participant text message responses. This process ensured a common, nuanced understanding of frames, themes and quotes and is a well-established approach for producing a high level of agreement (51).

#### Analysis of Focus Group Discussion Data

A full review of the transcripts generated from focus group discussions was conducted, from which short summaries of each transcript that was compiled. These summaries helped to identify the main themes and patterns across transcripts, highlighting intervention and platform utility among ALWH. In presenting our results, we identified themes that directly spoke to the ways in which mHealth interventions, as well as the methods and process employed, influenced intervention delivery.

## Results

Among 52 participants aged between 15 and 20 years enrolled in the study, 25 (56%) were female. Over 49 (94%) were retained in the intervention by the end of month 2, with 45 participants completing the exit surveys.

### Overall Use of the Technology Platform

Participants across both sites, regardless of age group, reported that the platform was “easy to use.” They reflected that the intervention and platform were acceptable, as indicated in the high levels of engagement (100% of participants sent at least one message). They reported that they enjoyed the fact that they could communicate “anytime, anywhere,” Many enjoyed communicating while on the bus to the clinic or on their way home from school and reported experiencing few network coverage issues. Participants liked that group facilitators monitored the group conversations primarily because they were able to respond to support needs of participants, troubleshoot technological issues, and uphold confidentiality.

### Privacy and Confidentiality Provided by the Platform

Participatory processes conducted at the beginning of the intervention showed that participants were concerned that they would be judged when accessing SRH services and when discussing intimate issues such as disclosure and adherence. They were receptive to the idea of a platform where they could discuss such topics anonymously and keen to engage in groups with a mix of genders in order to gather different experiences without being face-to-face. Exit group discussions confirmed this, as participants reported that they were able to freely communicate with one another along with a trained facilitator and peer mentors because they could remain anonymous. It was also important for them that the platform was not accessible to their families and that the phone they had was secure and confidential.

### Engagement and Group Dynamics

Engagement within groups was high, with all the participants sending at least one text message. In total, participants sent 18,253 text messages over 3 months, covering a wide range of topics, with both short and long exchanges. Participants sent an average of 351 messages each, and four messages per participant per day. Participants mainly communicated in English, as they were encouraged not to use local languages so as to include all participants in discussions. This was most difficult in the Cape Town-based groups, where participants were inclined to use Xhosa.

Participatory discussions and the initial kick off conversations in the groups elicited a number of “ground rules” for how the virtual space should be curated. This included value alignment amongst participants so as not to alienate others, such as maintaining respect and care, forbidding dating between participants, the time of communication, and modalities of managing conflict. Importantly, facilitators played an active role in setting these ground rules at the beginning of the intervention, but then participants took them up themselves encouraging everyone to maintain the rules as summarised in text format below:

*Kind “Can we get those rules now please?”*
*Lebogang “Ok”*
*Messi “what rules?”*
*Kind “I know that one that says no dating and remain anonymous”*
(Peer to peer text conversations)

In other groups, participants spoke about keeping the information confidential to the group members, and to write legible and clear text. They felt that these ground rules helped them to own the space. They called Khuluma their “*space to be.”*

In general, post intervention group discussions and text messages also illuminated the kinds of learnings obtained from peer-to-peer support groups or one-on-one text messages. These were learnings about living with HIV, messages of empathy, sympathy, and encouragement. As such they felt more open to share and accept their status as noted by one of the participants:

“*I learned that people living with HIV can do what people not living with HIV can do. No one had ever asked me to talk about my feelings in this way before. As I opened up more I learned to trust and could share more”* (Post intervention group discussion).

“*I learned that I should not be afraid to talk about my feelings”* (Post intervention group discussion).

Another participant highlighted that

“*Khuluma is the best thing in my life… I learned more in this group I love this group… everything I didn't understand mn [the facilitator] was there to make me to understand it. And I thank mn [the facilitator] and other mentors to give us this opportunity cause [because] they welcomed us with a great heart. There [They] are patient to [with] us they be [are] there when you need them always they make a problem to go [go away] like you didn't have it”* (peer-to-peer text message).

### Overarching Themes Emanating From the Content of Text Messages

When coded, messages included three overarching themes, namely: (i) social support, (ii) stigma and disclosure, (iii) medication and adherence (see Tables 2, 3).

**Table 2:** Text conversations on social support from facilitated group and one-on-one text messages.

**Main theme**	**Sub-themes and summary of discussion points**	**Sample text**
	**Informational support**	
**Social support**	***What makes the viral load to increase?** (Question posed by facilitator)*.	“Does everybody know what the viral load is & is your doctor happy about your blood results? Why is it that makes doctors to be unhappy with your blood results?”*. (**AN, group facilitator)***
	***Poor adherence to treatment** (participant response)*	“It is because u don't adhere to your treatment that can cause your treatment to be [resistant]. That [means] treatment [doesn't] works in your body because of those silly mistakes”… ***(Phumula, group participant)***
	***What makes our doctor unhappy?***	“I [think that the thing that makes] our doctor [not] happy is [when] u don't drink ur med every day and ur [your] viral load is going up or cd4 is going down”… ***(Promise, group participant)***
	**Conveying emotional support to one another**	
	***Worries about death from HIV***	“When I have HIV I am going to die?”… ***(Dimpo, group participant)***
	*Regular use of treatment will help*	“Mpumi no you won't die Remember that there is treatment available to help you live a long happy productive life,… ***(MN, group facilitator)***
	*Continues to worry*	“I think I am going to die”… ***(Dimpo, group participant)***
	*Re-assured*	“No Mpumi you are not going to die, yes there is no cure for HIV but there are so many people that have been living with HIV for many years”… ***(MN, group facilitator)***
	***Worries are prevalent in the group***	“Like us, you are not the only one”… ***(SM, group mentor)***
	*Relieved she is not alone*	“I was thinking am the only one”… ***(Dimpo, group participant)***
	***Use of medication emphasised***	“As long as you take your Medication as directed by the Doctor and also make sure that you discuss all your concerns with a doc”… ***(MN, group facilitator)***
	***A family of similar other***	“We are the same and we support each other and love each other”… ***(SM, group mentor)***
	*Words of support and togetherness*	“No you are not the only one Mpumi. We as Khuluma group are here for you and every person in this group is living with HIV”… ***(MN, group facilitator)***
	*Encourage love*	“Yes I agree with you we mast [must] love people”… ***(Dimpo, group participant)***
	*Relief she is will be ok*	“No I understand”… ***(Dimpo, group participant)***
	**Network support**	
	*Morning check-in by members of the group*.	“Good morng [morning] Prom. Dd u slp well!!! [Did you sleep well]”… ***(GMK004, group participant)***
	***Support from members of the group, now***	“Morning All Gyz: [guys]”… ***(NikiMinaj)***
	***a network of friends, a family other***	“Hw r u ben?[How have you been?]”… ***(Promise, group participant)***
		“Morning guyz”… ***(Phumula, group facilitator)***
		“Guys am going to church chat later hv [have] a nice day”… *(**Promise, group participant)***

**Table 3:** Text conversations on stigma, disclosure, and treatment adherence.

**Main Theme**	**Sub-themes and summary of discussion points**	**Sample text**
**Stigma and Disclosure**	***Worries about HIV***	“Ok, can anyone give others some advice for how to manage HIV?”*. **(MN, group facilitator)***“They must not be worry about HIV cause they are others who has [have] it like us”… ***(SM, group mentor)***
	***Labelled as slow minded due to treatment***	“But in life why other people say that the people who take ARV medication are slow in their minds, Why?”. ***(TK, group participant)***
	***Regarded as abnormal***	“Most people with HIV doesn't [don't] look [normal], they have skin changes and lips changes”… ***(anonymous1, group participant)***
	*Group provides reassurance*	“Because they told themselves that. but u know it's not true. They might say that but that is not true. We have many brilliant and smart people who are taking the medication all over the world”… **(KE, group participant)**
	**Participants found comfort in virtual groups to seek information around partner disclosure**	
	***When to disclose to a partner?***	“If u have a partner wen [when] is da [the] right to tell him or her that u r HIV?”… ***(Pronto, group participant)***
	*Seeks clarification*	“I don't understand plz [please] tell me”… ***(Angel, group participant)***
	*Repeats*	“If u love someone when is it the right to tell them?”… ***(Pronto, group participant)***
	*Participant advice*	“I will say baby I have HIV plz [please] don't cry”… ***(Angel, group participant)***
	***Mentor's advice***	“Me I will advise you that do what you think is right”… ***(SM, group Mentor)***
	*Another participant attempt at a response*	“I would say u [you] might not like me an more but I'm HIV [positive]”… ***(Pronto, group participant)***
		“*If someone love you she stay with u”*… ***(Angel, group participant)***
	**Let's talk about taking ARVs**	
**Treatment adherence**		“I feel like I'm a fool in everything. Like I think I'm a failure maybe for taking medication and I'm a fool. Now I need guidance”… ***(TK, group participant***)
	*Advice on medication use*	“But you know that it is not true.you are not a fool but you are a strong person who have been doing it right all this time. Taking your medication does not make you a fool in any way. Do you understand the importance of taking the medication?”. ***(AM, group facilitator)***
	***Medication makes one stronger***	“Ya! The medication that you take makes you stronger than you thought. It kills the weakness in you”… ***(KE, group participant)***
	***Adherence is needed***	“I agree with you Krazy E.because not taking your medication is the bad thing to do in this case”… *(**AM, group facilitator)***
	*Checking in on others members*	“Where is the rest of the group members? Good question hey. we need different views guys!”… ***(AM, group facilitator)***

#### Social Support

Participants reported providing and receiving three kinds of support—informational, emotional, and network support. Informational support involved text messages sharing/requesting information to help participants better understand a complex issue or situation that they may be facing. These were often related to sexual and reproductive health information or HIV-related information including opportunistic infections and onward transmission. Issues requiring clarification were usually brought up by the facilitator through the use of a question for participants. Facilitators also encouraged participants to bring up questions they had themselves and verify information where necessary (see Table 2).

Emotional support involved messages aimed at helping someone feel better in their situation, messages expressing/describing emotions and messages expressing empathy, sympathy and encouragement. These messages were often related to feelings about HIV positive status, feelings about the future and issues related to relationships (see Table 2). Messages of encouragement were common with some participants mentioning that communicating with others had helped them to think more positively about living with HIV and to feel that they had a future as described in the text message below:

“*I want us to pull up our souks [socks] and look forward not backward and we must not let hiv to overpower us”* (peer-to-peer text message).

“*Good nyt [night] believe in urself [yourself] and all tht [that] u Knw [you know] there is something inside u tht [you that] is greater than any obstacle”* (peer-to-peer text message).

Finally, participants provided each other with network social support, that included messages affirming a sense of belonging, companionship and solidarity; messages which sought to normalise experiences and overcome feelings of abnormality; expressions of gratitude to the group and checking-in messages (e.g., Hi, how are you doing?, what are you doing?) (see Table 2).

#### Stigma and Disclosure

Participants expressed desires to engage in friendships, make friends and increase their support network. The fact that they shared similar experiences was crucial to this, as one participant noted:

“*I feel more comfortable talking to people in this group because we all have HIV”* (peer-to-peer text message).

Trust and relationships were common topics, potentially because ALWH not only had to navigate the usual complexities and insecurity of adolescent relationships, but they also had the added complication of an illness that is socially stigmatised and transmittable. They noted how challenging it was to disclose their status to a potential partner or a friend, unlike on this platform.

“*U cn cht wth frnds bt dnt tll [you can chat with friend but don't tell] them secrets”* (peer-to-peer text message).

Participants further discussed sharing their HIV status and the fears they had about this to family, friends, and partners. They indicated that stigma and discrimination was the primary reason that they did not feel comfortable sharing their HIV status. Some expressed discomfort at hearing other people talk negatively about people living with HIV as reported below:

“*Most people with HIV doesn't [don't] look [normal], they have skin changes and lips changes”* (peer-to-peer text message).

“*They laugh at them and say all dis [this] negative things… Hiv pple [people with HIV are] disgusting. I feel scared: … sometimes I feel like nobody can talk with me or have fun with me”* (peer-to-peer text message).

Participants expressed strong fears that status sharing would lead to rejection, abandonment and discrimination. The word “hate” was frequently used to describe how they believed others would feel about them if they shared their status.

“*The hard part about living with hiv [HIV] is the fact that u have it and u r [you are] scared that someday people might* ***hate*** *u because u have hiv [HIV] and ur [you're] also scared that u might say it and then be ashamed”* (peer-to-peer text message).

Through the peer-led mHealth platform, many were able to ask questions of their peers experiencing the same challenges and obtained a variety of responses, as if they were chatting to a group of friends in a regular adherence support club, except in this context they could ask questions in comfort and confidentiality. In particular they were often concerned with sharing their status in the context of a romantic relationship, as one participant shared in the text below

“*Guys am positive neh what's going to happen when [I] am having sex with someone?”* (peer-to-peer text message).

Table 3 shows more examples of the kinds of discussions participants shared. Some participants expressed a desire to share their status, but also expressed concerns that this could be risky.

*I am afraid to tell my friends I am hiv, I can't tell my friends about my life* (peer-to-peer text message).

#### Medication and Adherence

The intervention impacted behaviours beyond the groups, most specifically around medical adherence. Here messages focused on sharing their own experiences or strategies for dealing with taking their medication such as:

“*My granny always makes sure I drank [drink] my medicine [medicine] and I am always healthy”* (peer-to-peer text message).

“*Me what I experience is that hiv is not a killer disease [it] is like flu so if you don't want people to see that you are hiv you must drink your treatment well don't skip a day and all the days your cd4 count must be up not down and your viral load must be down not up”* (Peer-to-peer text message).

“*Medication is lyk [like] bathin [bathing] or eatin, [eating] something u shouldn't forget. Make that promise to ur self n u wil [and you will] never forget to take ur meds”* (Peer-to-peer text message).

Participants discussed the side effects of taking medication and admitted to missing their medication due to these side effects.

*Me last year I was sick at school I drinked [drunk] my arv before eating so I learn a lesson never drink my tablet without eating* (peer-to-peer text message).

Taking medication seemed to be intricately connected to the other negative emotional states, particularly anger and depression, with reports that medication reinforced that they were somehow “different” from others. Reasons cited for missing medication (dose-skipping) included fears of disclosure and stigma.

“*No [one] can c [see] me [take my meds] n [and] think im HIV [positive] so to me i tell myself that im not HIV [positive] but then when it comes to takin meds im serious even if i go to my pals place”* (peer-to-peer text message).

In general, participants often reminded each other to take their medication, and shared strategies for dealing with side-effects and how to integrate a treatment regimen into their usual routine. Finally, the group setting allowed participants to discuss different perspectives on issues pertinent to their needs (Table 3). Discussing their relationships with a group that shared similar experiences and receiving relevant advice was important, especially as the anonymity allowed them to express their feelings freely.

## Discussion

This pilot study demonstrated that peer-to-peer, group spaces delivered *via* text message are feasible for the provision of social support, especially when they use “bottom-up” approaches that include participatory approaches to design, implementation and evaluation (18, 53–55). Such processes are critical for the rapid implementation of technological architectures, ensuring buy-in from participants, establishing social norms and behaviours, discouraging conflict and misinformation, and encouraging participants to share experiences. For adolescents in particular, while they are responsive and enthusiastic about mHealth interventions and mobile technology in general (56), curating a virtual space that fits their needs and tailoring it to their specifications are important for successful engagement. Indeed, for such participation in virtual spaces to be authentic, it must encourage ownership of that particular space by participants. It is through this sense of ownership that participants are able to develop a critical consciousness toward their condition and to feel empowered to make changes in their lives (57, 58). Within this context, we see authentic participation and ownership of virtual space, born through participatory intervention design and lived on through peer-led facilitation, as having important flow-on effects in allowing participants to open up about issues such as stigma and discrimination, thus changing adherence behaviour, sexual risk behaviour and encouraging participants to disclose their status to their peers.

A second factor that was critical to the successful curation of the virtual space was the development of a technological platform that fitted the behaviours and digital literacy of the study population. It was apparent from the participants' engagement during the pilot and from the exit group discussions that the use of phones and SMS technology was a moderately acceptable alternative platform for intervention delivery, comparable to in-person and in-facility engagements. The intervention design used technology as an enabler, not a solution, yet for the majority of participants, the platform provided intimate, accessible psychosocial support that could be engaged with “anytime, anywhere,” overcoming both logistical barriers to accessing support and social barriers of stigma. Creating a space confidential enough to allow participants to freely express their needs among others was critical to participants using this platform. This correlates with other adolescent interventions, where maintaining a free and non-judgemental space has been shown to be critical (10, 59). Further, it was important to provide participants with a new, study phone in order to ensure confidentiality, monitor and top up text messages. This is an important consideration in any scale up plan for these types of interventions.

The third factor was the use of active facilitation that involved a combination of professional experts and peer mentors. The role of peer-led counselling in SRH is growing in importance in Sub-Saharan Africa (SSA), as evidenced by a range of important interventions that involve youth-friendly spaces such as in-community or in-clinic hubs (60) or adherence clubs (61). This intervention confirmed the value of peer support in virtual spaces. The combined approach of having both trained counsellors to provide accurate information, difuse conflict, and manage difficult conversations; and peer mentors who shared the experiences of the participants, was critical in developing a space that was safe and welcoming, but also efficacious in improving HIV and treatment adherence, knowledge and support. The leadership provided by peer mentors and tone of the peer-led conversations allowed participants to overcome stigma and feel less isolated in their experiences of living with HIV. This was critical to them opening up about their experiences, and feeling motivated to develop new skills and accept new information about their condition. Hence, they were able to discuss intimate topics like disclosure and adherence without feeling judged. It was critical that this space was not dominated by information provided by health care professionals or elders, but instead balanced, informational, peer-led and emotionally supported.

Finally, it was important that group conversations did not, and were encouraged not to, only focus on HIV. Participants' conversations ranged topics including relationships, education, home-environments, entertainment, and careers. Though they had a common experience of living with HIV, this did not dominate, but mediated deeper conversations about stigma, disclosure, and adherence. This shows that ALWH lives are not defined only by their positive status, highlighting once again the importance of peer support groups (22, 23). It was important that participants were allocated or moved to a group that was discussing experiences pertinent to theirs. It was only through the provision of this level of support that participants felt that they were able to open up about more intimate issues.

### Study Limitations

Not all participants' needs can be met through this technology and referral pathways in these instances are critical. In addition, while young people may have access to mobile phones, they have limited access to data. More recently, the increase in Wifi-free zones or “hotspots” is proving to be useful for many adolescents without data and might see increased utilisation of mHealth interventions. The model, however, used a basic SMS technology, which may work for small group facilitation but which may not work for larger groups. Larger groups may require more sophisticated monitoring functionality. Such an increased degree of monitoring functionality has been achieved through the transition of the platform in recent years to android smart phones and the Rocket.Chat platform. The technology is therefore available but access to this technology amongst the target population remains varied, albeit increasing. Our study worked with adolescents most of whom are attending school. Some were unreachable especially when attending to school activities.

## Conclusion

The role of virtual spaces in the provision of psychosocial support was shown to be more critical than ever before, due to restrictions on social interaction imposed as a result of COVID-19 lockdowns. This has also been proven for many health services including adolescent SRH services, patient follow-up and, medication adherence support services. This paper has highlighted a set of processes central to the successful implementation of an mHealth intervention. These include but are not limited to: the use of participatory methods in intervention design, the development of a technological platform that meets the digital literacy needs of the population, the provision of peer-led facilitation, and the development of diverse topics to curate an effective virtual space that can support young people living with HIV. Mobile phone-based support interventions are especially useful among adolescents needing safe spaces to freely express themselves and to provide support for each other.

## Data Availability Statement

The original contributions presented in the study are included in the article/supplementary material, further inquiries can be directed to the corresponding author/s.

## Ethics Statement

The studies involving human participants were reviewed and approved by Human Sciences Ethics Committee at the University of Cape Town (HREC 630/2014) and the University of Pretoria (GW20160616HS), including the protocol, informed consent documents and tools. Written informed consent to participate in this study was provided by the participants' legal guardian/next of kin.

## Author Contributions

MA and NS: abstract writing and full manuscript writing including methodology, analysis, results, discussion, and conclusion. MN and TM contributed to adding sections in the study implementation processes. CP, DG, and ZF: review and editing of the manuscript. L-GB, MV, MB, and AK: protocol leads and final review. All authors revised and approved the final draft.

## Conflict of Interest

The authors declare that the research was conducted in the absence of any commercial or financial relationships that could be construed as a potential conflict of interest.
